# Resource quality affects weapon and testis size and the ability of these traits to respond to selection in the leaf‐footed cactus bug, *Narnia femorata*

**DOI:** 10.1002/ece3.2017

**Published:** 2016-02-26

**Authors:** Daniel A. Sasson, Patricio R. Munoz, Salvador A. Gezan, Christine W. Miller

**Affiliations:** ^1^ Whitney Laboratory for Marine BioscienceUniversity of Florida9505 Ocean Shore BlvdSt. AugustineFlorida32080; ^2^ Department of AgronomyUniversity of Florida2005 SW 23 St. Bldg 350GainesvilleFlorida32611; ^3^ School of Forest Resources & ConservationUniversity of FloridaPO Box 110410GainesvilleFlorida32611; ^4^ Entomology and Nematology DepartmentUniversity of FloridaPO Box 110620GainesvilleFlorida32611

**Keywords:** Development, evolvability, heritability, *Narnia femorata*, nutrition, sexual selection, testes, weapons

## Abstract

The size of weapons and testes can be central to male reproductive success. Yet, the expression of these traits is often extremely variable. Studies are needed that take a more complete organism perspective, investigating the sources of variation in both traits simultaneously and using developmental conditions that mimic those in nature. In this study, we investigated the components of variation in weapon and testis sizes using the leaf‐footed cactus bug, *Narnia femorata* (Hemiptera: Coreidae) on three natural developmental diets. We show that the developmental diet has profound effects on both weapon and testis expression and scaling. Intriguingly, males in the medium‐quality diet express large weapons but have relatively tiny testes, suggesting complex allocation decisions. We also find that heritability, evolvability, and additive genetic variation are highest in the high‐quality diet for testis and body mass. This result suggests that these traits may have an enhanced ability to respond to selection during a small window of time each year when this diet is available. Taken together, these results illustrate that normal, seasonal fluctuations in the nutritional environment may play a large role in the expression of sexually selected traits and the ability of these traits to respond to selection.

## Introduction

Male–male competition over access to females is common across animal taxa and has driven the evolution of enlarged male weapons. In many cases, those males with the largest weapons are predicted to achieve more mating opportunities (Andersson 1994). Interestingly, the size of weapons can vary tremendously within natural populations. The size of weapons is often highly influenced by the nutrition that males experience during weapon formation (Moczek and Emlen 1999; McLain and Pratt 2010; Emlen et al. 2012), but additive genetic variation can also contribute to weapon size (e.g., Kruuk et al. 2002; Gotoh et al. 2012). Although weapon size can be important, reproductive success is not determined by weapon size alone. Female mate choice can be important even in species with male–male competition (Hunt et al. 2009; Miller and Svensson 2014). Furthermore, sexual selection does not end at mate acquisition in many systems. Postcopulatory sexual selection, via, for instance, sperm competition, can have a large impact on an animal's reproductive success (reviewed in Birkhead and Pizzari 2002 and Snook 2005). Despite this realization, only a few studies have evaluated the genetic and environmental influences on the expression of traits important for postcopulatory reproductive success (e.g., Engqvist 2008; Lewis et al. 2012). Moreover, these studies have often been conducted in static or unrealistic settings which make it difficult to generalize their results to natural systems. Resource availability and quality during development often impact the size of adult traits (e.g., Moczek 1998; Karino et al. 2004), and they can also influence the heritability and evolvability of traits. Heritability may be particularly likely to change as environments become more stressful; however, no clear pattern has emerged as to whether stressful environments increase or decrease trait heritability (reviewed in Hoffmann and Merilä 1999). Heritability may increase in stressful environments when, for example, differences in genotypes become apparent in response to the limited food (Hoffmann and Merilä 1999). Alternatively, stress may limit the expression of genetic variation, leading to lower heritabilities in stressful environments (Hoffmann and Merilä 1999).

The majority of studies into the role of genes and the environment have focused on either precopulatory or postcopulatory sexually selected traits (e.g., Qvarnstrom 1999; Miller and Brooks 2005; Mills et al. 2007; Greenfield et al. 2012). However, traits that influence precopulatory and postcopulatory reproductive success do not develop in isolation. Animals have limited resources and must allocate those resources between the soma and the gametes (Stearns 1989). Precopulatory traits are often governed by somatic tissues, while postcopulatory traits are associated with the gametes. Thus, physiological decisions to allocate resources between the soma and the gametes may affect the expression of precopulatory traits at the expense of postcopulatory traits or vice versa. Examples of such differential allocation decisions have been found across taxa (e.g., Simmons and Emlen 2006; Thomas and Simmons 2009; Evans 2010; Rowe et al. 2010). In this study, we investigate the contribution of genetic and environmental factors to the expression of traits linked to precopulatory and postcopulatory reproductive success in the leaf‐footed cactus bug.

**Figure 1:**
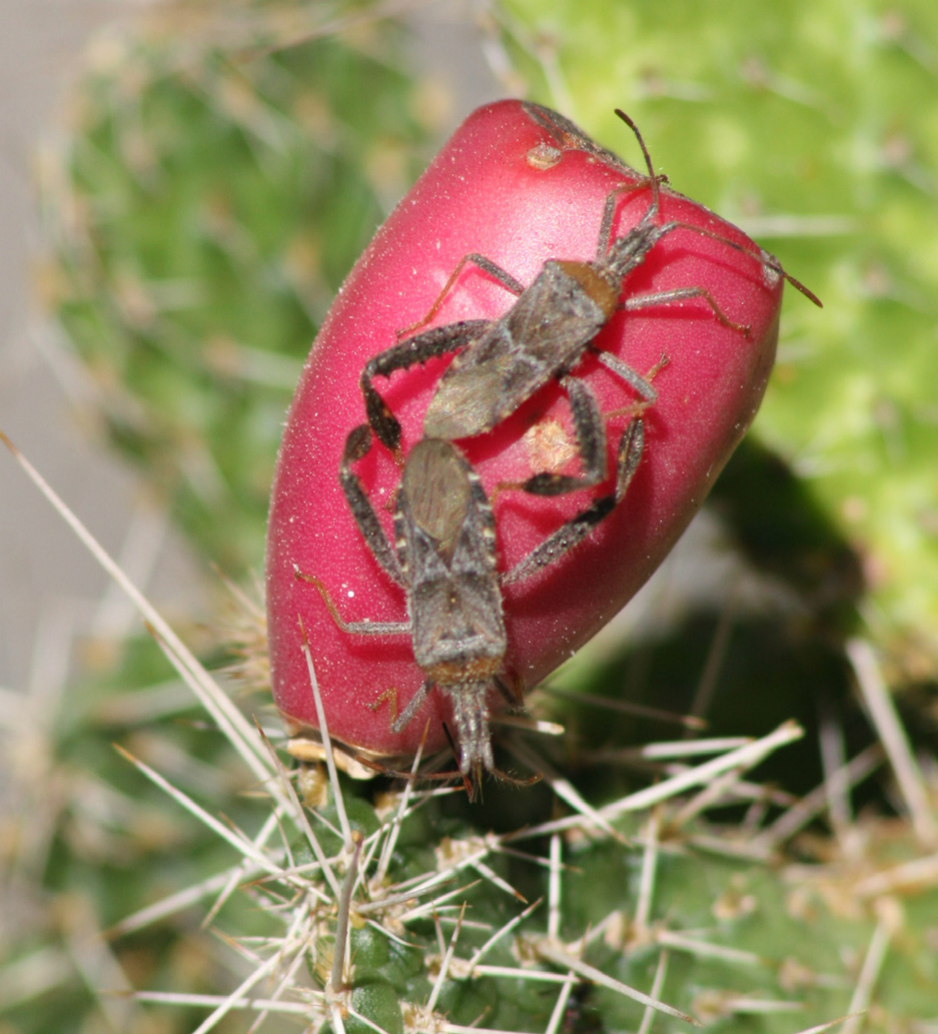
A pair of *N. femorata* mating on ripe fruit of the cactus *O. humifusa*.

**Figure 2:**
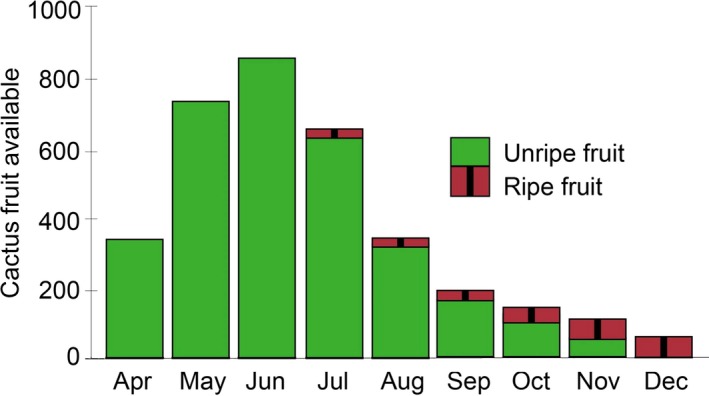
*Opuntia humifusa* fruit availability and quality from April to December 2010. Data collected from a 160‐m‐long, one‐meter‐wide transects at the Ordway‐Swisher Biological Station. Figure modified with permission from Gillespie et al. (2014).

*Narnia femorata* (Hemiptera: Coreidae, Fig. 1), the leaf‐footed cactus bug, was introduced to Florida from the Southwestern United States in 1960s (Baranowski and Slater 1986). In North‐Central Florida, the bug lives and feeds mainly on the cactus plant *Opuntia humifusa*, using its beak to suck out the juices from the cactus fruit and pads (P. Allen and C. Miller, in prep.). Cactus fruit is an ephemeral resource; cactus fruit availability changes spatially and throughout the year (Fig. 2, Gillespie et al. 2014). Ripe fruit is only available for a short period of time before it is rapidly depleted by herbivore competitors (González‐Espinosa and Quintana‐Ascencio 1986; Hellgren 1994). Juvenile *N. femorata* lack wings and are not highly mobile; thus, they are limited to the resources available in the area where they hatch. As a result, they may have ripe cactus fruit available for only part of their development before it is removed by deer, birds, raccoons, and other cactus‐fruit‐eating competitors. Previous work has shown that bugs raised on cactus without fruit for part or all of the development grow more slowly and reach smaller sizes as adults than those raised on cactus with ripe fruit (Nageon de Lestang and Miller 2009; Gillespie et al. 2014).

Adult male *N. femorata* compete with other males for access to cactus territories using their hind femurs, a sexually dimorphic feature highly correlated with body size (Procter et al. 2012). Larger males are more likely to win these territorial contests (Procter et al. 2012; Z. Nolen, in. prep.) and are thus more likely to encounter females who search for high‐quality cactus on which to mate and lay their eggs. Female *N. femorata* prefer to mate with large males (Gillespie et al. 2014); thus, males successful in territorial contests likely have a sizable mating advantage.

Female *N. femorata* often mate with multiple males (C.W. Miller, unpublished data) and can store sperm in their spermathecae (D. Sasson, unpublished data). Thus, postcopulatory sexual selection is probably an important factor in male reproductive success. Success in postcopulatory sexual selection may be especially important for small males, who probably achieve far fewer opportunities to mate than larger males (Gillespie et al. 2014). Therefore, the level of sperm production may be an important facet of postcopulatory reproductive success in *N. femorata*. Sperm competition has been documented in other Hemipteran species (e.g., Rubenstein 1989; Carroll 1991; Garcia‐Gonzalez and Gomendio 2004), and testes mass positively correlates with sperm number in *N. femorata* (P. Joseph, unpublished data). Males with larger testes likely can transfer more sperm to females which may increase their postcopulatory reproductive success (P. Joseph in prep.).

In this study, we examine the genetic and environmental effects on femur and testis size in *N. femorata* and how developmental environment may affect resource allocation decisions across these sexually selected traits. We address three objectives: (1) Whether the genetic variation, heritability, and evolvability of femur and testis size change across the developmental environments. Work in this species and closely‐realated species has demonstrated that males with poor nutrition are smaller overall (Miller 2008; Miller and Emlen 2010; Gillespie et al. 2014). Thus we expect that males raised on cactus with ripe fruit will (2) have absolutely larger femurs and testes than those raised without ripe fruit. In addition, we tested (3) whether the scaling relationships of femurs and testes change across the developmental environments. Precopulatory and postcopulatory traits may be linked through resource allocation decisions (e.g., Simmons and Emlen 2006; Rowe et al. 2010), and the quality and quantity of resources during development may affect these allocation decisions. We predict that these scaling relationships will differ between males raised in high‐ and low‐quality environments. In particular, because males from low‐quality environments may be more reliant on postcopulatory mechanisms for reproductive success, we anticipate that these males will have larger testes relative to weapon size than males from high‐quality environments. We addressed these questions using a half‐sib split‐brood experiment with insects raised in three developmental environments: cactus pads without fruit (low quality), cactus pads with unripe fruit (moderate quality), and cactus pads with ripe fruit (high quality). We used these ecologically relevant developmental environments because an important goal of this study was to understand how sexually selected traits are expressed and evolve in natural settings.

## Material and Methods

### Animal husbandry

**Figure 3:**
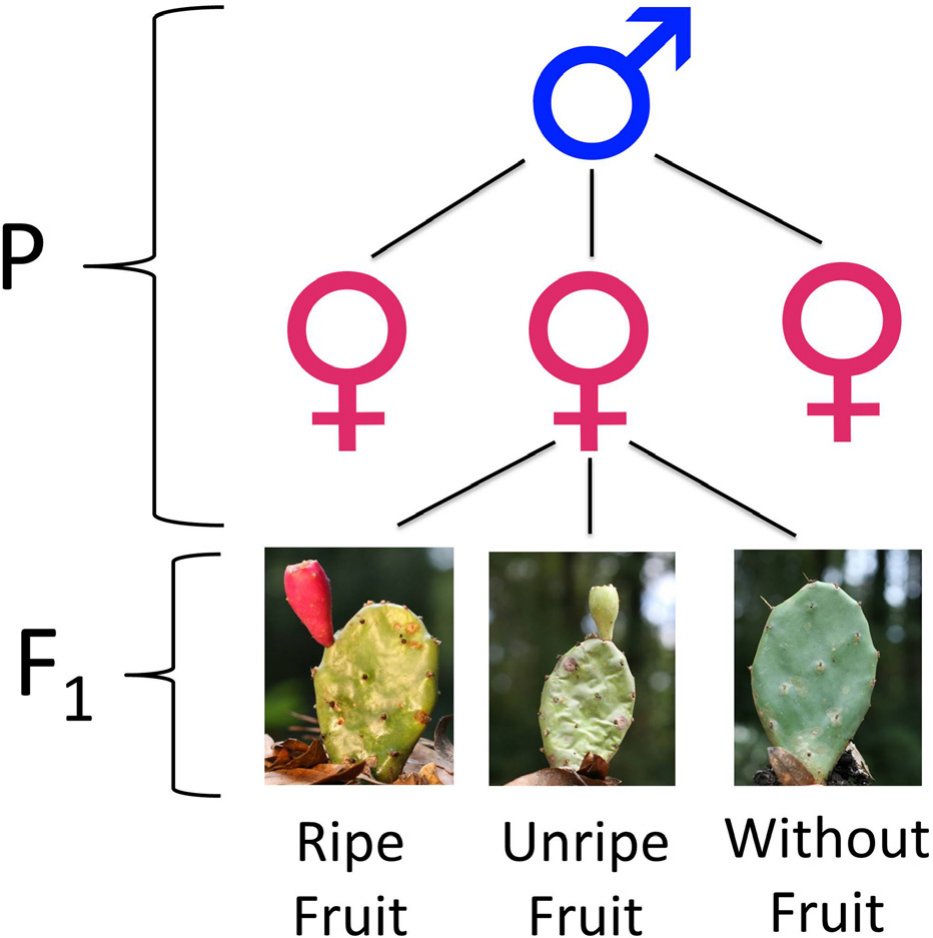
Schematic of the half‐sib split‐brood design. This schematic represents one sire family. Thirty‐four sire families were used in the experiment. Each male mated with three virgin females. The offspring of these three females were placed into the developmental environments with ripe fruit, unripe fruit, or without fruit at beginning of their 4th instar, and F_1_ males were the focal males in the experiment.

A colony of *N. femorata* was started at the University of Florida from adult bugs collected at the Ordway‐Swisher Biological Station (29°40′27″N, 82°1′40″W) in the fall of 2009. The insects used in this experiment are a subset of those bred as a part of a large, split‐brood rearing experiment. Adults from the 4th and 5th laboratory generation were used as parents of the focal bugs. Parental males (sires) were mated with three unrelated females (dams) (Fig. 3). Offspring of these three crosses were half‐sibs. These focal offspring were raised in small family groups of full‐sibs with access to *Opuntia humifusa* cactus bearing ripe fruit until they reached their 4th instar. *N. femorata* has five total instars. The first instar lasts only 2 or 3 days, and instars 2 through 5 each last approximately 1 week. *Opuntia* was collected at the Ordway‐Swisher Biological Station and Camp Blanding, a military facility 36 miles northeast of the University of Florida. Upon reaching their 4th instar, we gave each individual a unique identification number and randomly assigned them to their own container containing a cactus pad with whole ripe fruit, whole unripe fruit, or without fruit. Our methods mimicked the realistic situation where a competitive herbivore comes through an area, removing cactus fruit. Individuals were kept isolated in their container through the rest of their juvenile development. Fruits that began to dry out during this developmental period were replaced with fresh fruit. Upon reaching adulthood, every individual was sexed and transferred to a new container containing cactus with ripe fruit. Developmental environment is crucial to the expression of femur size, because once these insects molt to adults, their femur size is set for life. Testes mass, on the other hand, appears to be influenced by both developmental and adult conditions (P. Joseph, D. Sasson, P. Allen, U. Somjee, C. Miller, in prep.). We switched all insects to ripe fruit at adulthood to focus specifically on the effects of juvenile nutrition on adult attributes. This switch to a high‐quality diet at adulthood may mimic the natural progression of cactus fruit ripening as it occurs later in the year (Fig. 2) coupled with the ability of adults to fly to the best resources. Between 14 and 21 days after becoming adults, all but 74 individuals were a part of a larger experiment that allowed them to interact with other males and with females as a part of a larger experiment. Some of these interactions resulted in copulation. Two weeks after these behavioral trials ended, that is, 4–5 total weeks after reaching adulthood, we sacrificed the males to measure their femur, body, and testis size. The 74 males that were not in these behavioral trials only had their body and testis size measured. Their body and testis measurements did not significantly differ from those males used in behavioral trials, and so data from all males were included in the analyses.

### Measurements

We measured femur area for 910 males from 35 sire (half‐sib) families. We measured the body mass and testis mass, but not femur area, for an additional 129 males, for a total of 1039 individuals. Of the 35 sire families, 32 families had at least three individuals in each environment, and most had many more (median and range of individuals per sire family, cactus pads with ripe fruit: 11 [2–19], unripe fruit: 6 [0–15], without fruit: 14 [0–22]). Only 1 out of the 35 sire families did not have at least one individual from every environment, while 2 out of the 35 sire families only had 1 individual in the unripe fruit environment. Components of phenotypic variance were calculated using all sire families.

#### Femur area

First, we removed the femurs from the body and took pictures of each set of femurs under a dissecting microscope (Leica M165 C) using a digital camera (Canon EOS 30D). A photograph of a scale bar was taken before each set of pictures so that we could accurately measure femur dimensions. Using ImageJ (v1.42d, Abràmoff et al. 2004), we next outlined each femur and calculated the area of that outlined femur. We did this for both femurs and averaged the area of each male's femur for the measurement of femur size. Once the femurs were measured, insects were frozen until they could be dissected.

#### Body and testis mass

We placed the extracted testes on the preweighed aluminum foil that we then loosely folded and inserted into paper coin sleeves. The remaining body parts (including the femurs) were placed into another set of paper coin sleeves. We placed both coin sleeves in a drying oven set to 60°C and allowed all body parts, including femurs, and testes to dry for at least 7 days. After this period, we removed the testes from the oven and weighed each on a microbalance (Sartorius CP2P) to the nearest ng (minus the mass of the aluminum) to calculate the total dry mass of the testes. We then calculated the average testis mass for each male. Body dry mass (without the testes) was measured by weighing to the nearest mg on a balance (Sartorius 310S).

### Statistical analyses

#### Environmental effect on trait expression

We first analyzed the effect of natal environment on body mass, femur size, and testis mass. We log‐transformed all three variables to achieve normality before the analysis. For each trait, we ran an ANOVA with natal environment as the explanatory variable and the trait in question as the response variable. Post hoc analyses were conducted using Tukey's HSD (*α *= 0.05).

We used ANCOVAs to test the effect of the developmental environment on how femurs and testes scale relative to body size and relative to each other. We first compared how bugs from the three environments expressed femur size as a function of body size by running an ANCOVA with femur size as the response variable, environment as the explanatory variable, and body size as a covariate. To test how the environment affected testis size, we ran a similar model but with testis mass as the explanatory variable. Finally, we ran a set of three ANCOVA models to investigate the relationships among body mass, femur area, and testis mass. For these analyses, interaction effects between the environment and explanatory variables were removed from the models when *P* > 0.20 (Tomkins and Simmons 2002). Post hoc analyses were conducted using Tukey's HSD (*α *= 0.05). All analyses of the environmental effect on trait expression were conducted in JMP 11.0 (SAS Institute, Cary, NC).

#### Heritability and evolvability

Narrow‐sense heritability is measured as the proportion of phenotypic variation (*V*_P_) accounted for by the additive genetic variation (*V*_A_) (i.e., *h* ^2^ **= *V*_A_/*V*_P_). *V*_A_ and *V*_P_ can be specific to the environment in which they are measured, and differences in either (or both) *V*_A_ or *V*_P_ across environments can result in different measures of heritability and evolvability. We calculated the heritability across environments for body mass, femurs size, and testis mass separately, using the untransformed data. We fitted the following individual linear mixed model that considers the additive genetic relationships among relatives (based on pedigree) associated with each individual using the statistical program ASReml v.3 (Gilmour et al. 2009). The model fitted was as follows:

$$y=1\mu +X_{1}\tau +X_{2}\beta +Za\times \tau +e$$

where ***y*** corresponds to the response variable (i.e., body mass, femur area, or testis mass); *μ* is the overall mean; ***τ*** is a vector of fixed effects of the reared environment (ripe, unripe, and without fruit); ***β*** is a vector of fixed block effects of the person dissecting the insect; ***a×τ*** is a vector of random interaction effects between the additive individual genetic effect and the reared environment, with ***a×τ*** ~ MVN(**0**,***A***⊗***G***); and **e** is a vector of independent residual errors, with **e** ~ MVN(**0**,* σ²**I***). Additionally, ***1*** is a vector of ones; ***X***_*1*_, ***X***_*2*_, and ***Z*** are incidence matrices; and ***0*** and ***I*** are a vector of zeros and an identity matrix of its corresponding size, respectively. The matrix ***A*** corresponds to the relationship matrix derived through pedigree and to ***G*** is a 3 ***×*** 3 matrix of the unstructured form containing on the diagonal the variance components $\sigma_{aT1}^{2}$ , $\sigma_{aT2}^{2}$ , and $\sigma_{aT3}^{2}$, corresponding to the additive variance of individuals that have the environment groups ripe, unripe, and without fruit, respectively; the variance component of the off‐diagonal corresponds to *σ*_*aT*1*T*2_, *σ*_*aT*1*T*3_, and *σ*_*aT*2*T*3_, which represents the covariance between additive effects among environment groups. Individual males reared on different environment levels are connected with each other through their sires. Significance of fixed effect terms was obtained using an approximate F‐test together with the predicted mean values for each level.

The REML‐estimated variance components were later used to calculate the heritability associated with each environment (*i* = 1, 2, or 3, corresponding to ripe, unripe, and without fruit) grouped together with their standard errors (based on a delta method approximation) as follows:

$$h_{aTi}^{2}=\frac{\sigma_{aTi}^{2}}{\sigma_{aTi}^{2}+\sigma^{2}}$$

The short‐term evolvability of a trait depends on an additive genetic variation (Houle 1992), and so heritability has historically been used to describe how readily a trait can evolve. However, the improved estimates of evolvability are now available (Houle 1992; Hansen et al. 2011; Garcia‐Gonzalez et al. 2012). For this study, the coefficient of additive genetic variance (CV_A_) was calculated as √ *V*_A_/ $\overset{¯}{X}$ where $\overset{¯}{X}$ equals the trait mean (Garcia‐Gonzalez et al. 2012). CV_A_ is reported here without the common 100 multiplier (Garcia‐Gonzalez et al. 2012). *I*_A_, another measure of evolvability, was also calculated as *V*_A_ standardized by the trait mean squared (Houle 1992). *I*_A_ measures the proportional change in the trait expected after one unit strength of selection (Hansen et al. 2011).

## Results

### Effects of developmental environment on morphology

As expected, we found that insects raised on cactus pads with ripe fruit were larger and heavier than insects raised on unripe or without fruit.

#### Body mass

**Figure 4:**
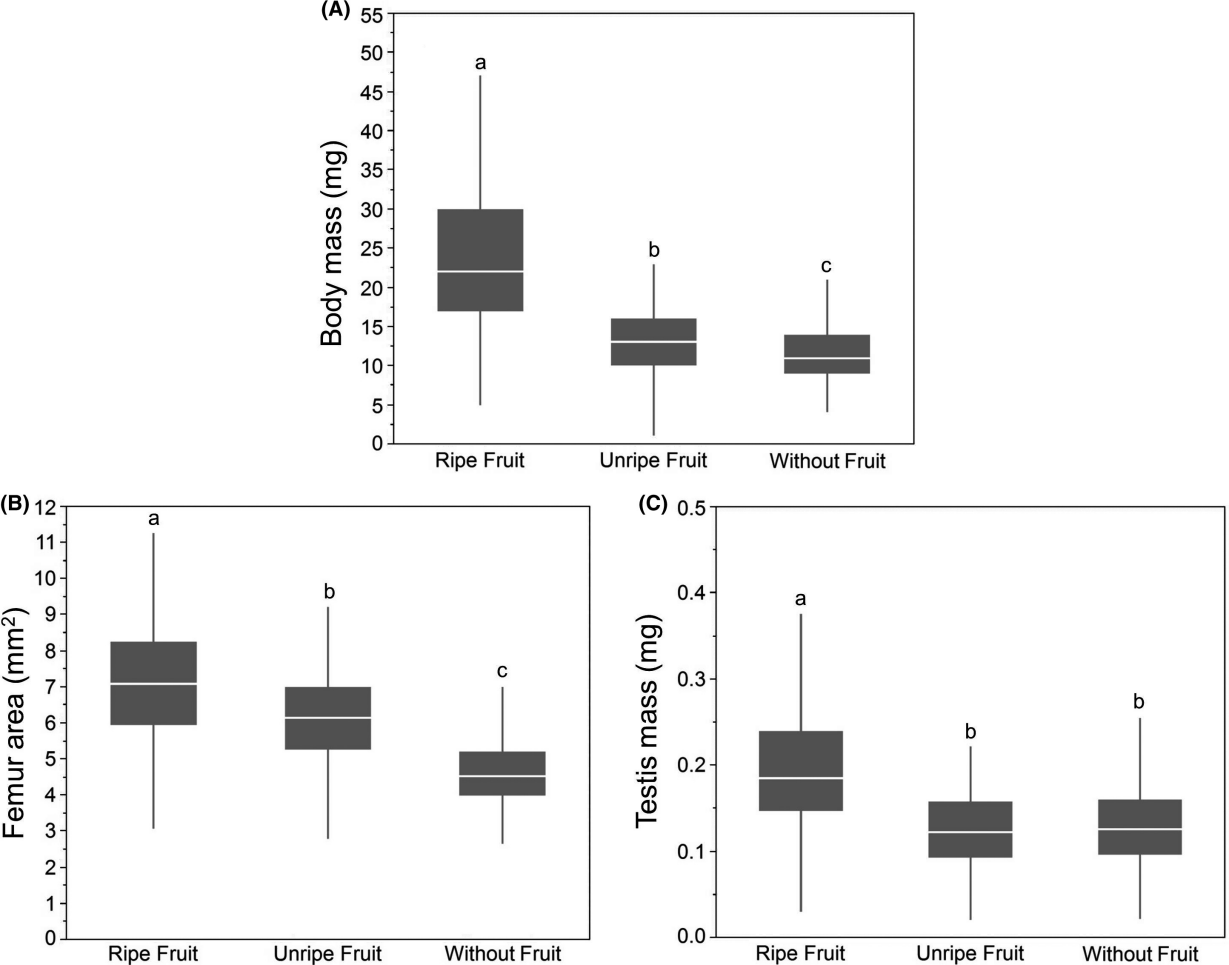
The effects of diet on body mass (A), femur area (B), and testis mass (C). For every trait, males raised on cactus with fruit were larger than males raised on cactus with unripe or without fruit. Lowercase letters indicate significant differences across the developmental environments based on Tukey's HSD (*α *= 0.05). Significant differences based on analyses using transformed data; untransformed data shown here.

Males that were raised on cactus pads with ripe fruit were larger than males from the other two developmental environments (Fig. 4A, ANOVA, *F*_2,1036_ = 282.5, *P* < 0.0001, Tukey's HSD, *P* < 0.0001), while those males raised on cactus pads with unripe fruit were larger than males raised on cactus pads without fruit (Tukey's HSD, *P* = 0.04).

#### Femur area

Males raised with ripe fruit had the largest femurs (Fig. 4B, ANOVA, *F*_2,899_ = 216.9, *P* < 0.0001), while males raised on cactus pads without fruit had the smallest femurs (Tukey HSD, *P* < 0.0001).

#### Testis mass

Males that developed on cactus pads with ripe fruit had larger testes than males raised on cactus pads with unripe fruit or without fruit (Fig. 4C, ANOVA, *F*_2,1036_ = 150.4, *P* < 0.0001, Tukey's HSD, *P* < 0.0001), while males on cactus with unripe fruit or without fruit did not differ from each other in testis size (Tukey's HSD, *P* = 0.60).

### Trait allometry across environments

#### Body mass and femur area

**Figure 5:**
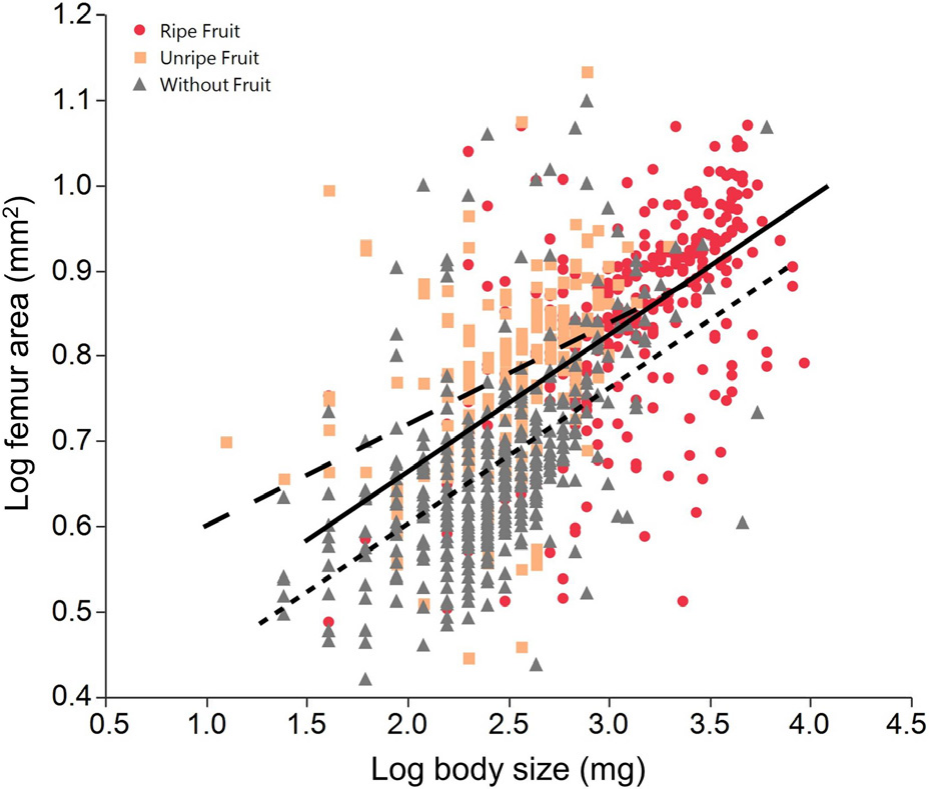
The scaling relationship between body mass and femur area. Males from the unripe fruit environment had the largest femurs relative to body size. Males raised without fruit had the smallest femurs at all body sizes. The solid regression line represents the ripe fruit environment, the long‐dashed regression line represents the unripe fruit environment, and the short‐dashed regression line represents the without fruit environment. Regression lines shown here were calculated from the transformed data rather than from the ANCOVA model.

We found a significant effect of the developmental environment on the scaling relationship between body mass and femur size (Table 1). Males raised on cactus without fruit had the smallest femurs relative to their body mass (Table 1), while males raised on unripe fruit had the largest femurs for their body size (Fig. 5).

**Table 1:** ANCOVA statistical results for scaling relationships between body mass, femur area, and testis mass. Bolded text indicates the overall model results for the two traits being compared in each ANCOVA. Nonbolded text indicates the statistical results for each effect in the model. In all cases, interactive effects were removed from models if *P* > 0.20

	Degrees of freedom	*F* ratio	*P*‐value
**Body vs. femur**	**3**	**190.0**	**<0.0001**
Body mass	1	275.1	<0.0001
Environment	2	50.7	<0.0001
**Body vs. testis**	**3**	**348.6**	**<0.0001**
Body mass	1	602.5	<0.0001
Environment	2	8.1	<0.001
**Femur vs. testis**	**3**	**135.1**	**<0.0001**
Femur area	1	102.5	<0.0001
Environment	2	45.0	<0.0001
Environment × femur area	2	1.9	0.15

#### Body mass and testis mass

Males raised on cactus pads with unripe fruit had the smallest testes relative to their body mass (Table 1, Tukey's HSD test, *P* < 0.01). Males raised on cactus with ripe fruit and without fruit did not differ in their relative testis mass (Tukey's HSD, *P* = 0.15).

#### Femur area and testis mass

**Figure 6:**
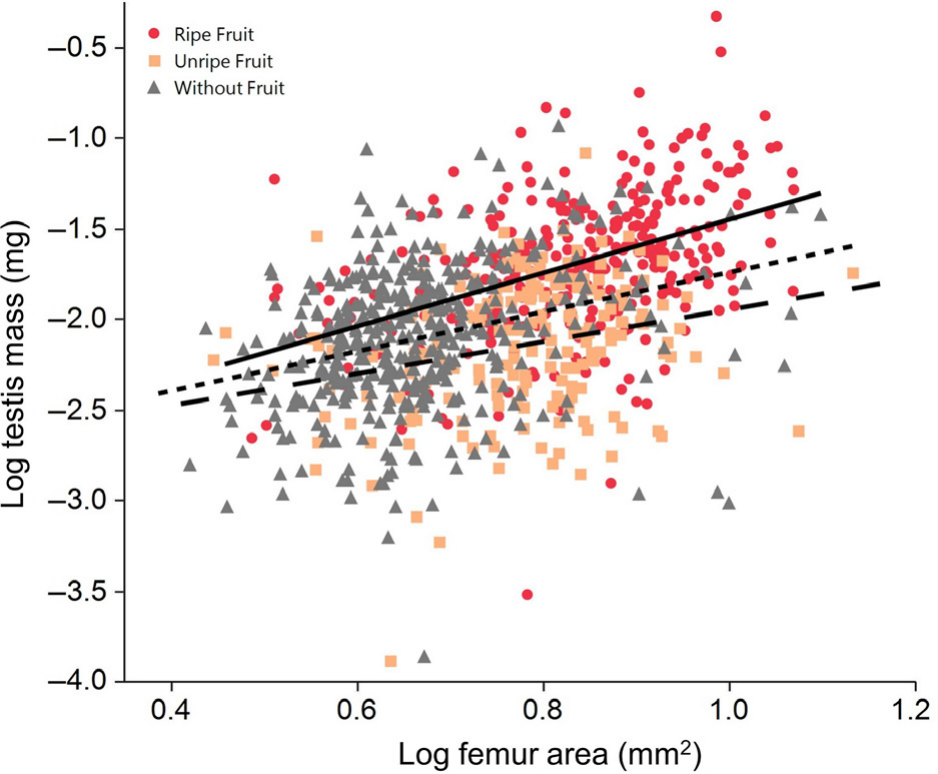
The scaling relationship between femur area and testis mass. We found no significant differences in the slopes of males raised in the three environments. Males raised on cactus with unripe fruit had smaller testes for their femur area than males raised on cactus with ripe fruit or without fruit. Males raised on cactus with ripe fruit had the largest testes relative to their femur area. The solid regression line represents the ripe fruit environment, the long‐dashed regression line represents the unripe fruit environment, and the short‐dashed regression line represents the without fruit environment. Regression lines shown here were calculated from the transformed data rather than from the ANCOVA model.

Males raised on cactus with ripe fruit had significantly larger testes relative to their femurs than males raised on unripe (Tukey's HSD test, *P* < 0.0001) or without fruit (Tukey's HSD test, *P* < 0.0001). Males raised on cactus with unripe fruit had significantly smaller testes relative to their femurs than males raised without fruit (Fig. 6, Table 1, Tukey's HSD test, *P* < 0.0001).

### Trait heritability and evolvability

The heritability of femur area was low in all developmental environments (Table 2). Body mass, testis mass, and femur area had heritabilities with standard errors that did not overlap zero in the cactus pads with ripe fruit environment, but did overlap zero in the cactus pads with unripe fruit environment (Table 2). Testis mass and femur area, but not body mass, had low heritabilities but with standard errors that did not overlap zero for those males raised without fruit. For all environments, *V*_A_, *V*_P_, CV_A_, and *I*_A_ were highest in the ripe fruit environment (Table 2).

**Table 2:** Trait means, additive genetic variation (*V*_A_), phenotypic variation (*V*_P_), the coefficient of additive variation (CV_A_), heritability (*h* ^2^), standard error of heritability (SE (*h* ^2^), and evolvability (*I*_A_) for testis, femur, and body size across diets of cactus with ripe fruit, unripe fruit, and without fruit

	Cactus with ripe fruit	Cactus with unripe fruit	Cactus without fruit
Body mass			
Mean (mg)	23.51	12.96	12.29
*V*_A_	36.53	0.97	1.51
*V*_P_	90.68	15.87	20.63
CV_A_	1.56	0.07	0.12
*h* ^2^	0.40	0.06	0.07
SE (*h* ^2^)	0.13	0.11	0.07
*I*_A_	0.07	0.01	0.01
Femur area			
Mean (mm^2^)	7.14	6.17	4.86
*V*_A_	0.41	0.0	0.26
*V*_P_	3.09	1.78	2.12
CV_A_	0.06	0.00	0.05
*h* ^2^	0.13	0.00	0.12
SE (*h* ^2^)	0.11	0.00	0.08
*I*_A_	0.01	0.00	0.01
Testis mass			
Mean (mg)	0.20	0.13	0.13
*V*_A_	1.08	0.16	0.26
*V*_P_	5.08	1.79	2.49
CV_A_	5.40	1.23	2.0
*h* ^2^	0.21	0.09	0.11
SE (*h* ^2^)	0.11	0.12	0.07
*I*_A_	27.00	9.50	15.40

## Discussion

In this study, we used nutritionally realistic environments to investigate the role of genes and developmental environment on body mass and two traits likely important in male reproductive success – femur size and testis mass. We found that the developmental environment had a profound effect on body mass, femur area, and testis mass (Fig. 4). In particular, males raised on cactus with ripe fruit had larger femurs (Fig. 4) and testes than males raised without fruit. These results support existing research showing the large effect of nutrition on size and development in this species (Nageon de Lestang and Miller 2009; Gillespie et al. 2014; P. E. Allen, in. prep.). We found that males reared on cactus pads with unripe fruit were bigger and had larger femurs than males reared on cactus pads without fruit (Fig. 4), suggesting that unripe fruit contains resources not available in the cactus pads. Larger males have an advantage when competing for females, at least in some contexts (Procter et al. 2012; Gillespie et al. 2014;), so the developmental environment experienced by juveniles may play a significant role in future male reproductive success.

Additive genetic variation, heritability, and evolvability were generally low for all three traits (Table 2); however, we did find evidence that the developmental environment influences these measures. The heritabilities of body and testis mass were highest for males raised on cactus pads with ripe fruit. Both total phenotypic variation (*V*_P_) and additive genetic variation (*V*_A_) for body and testis mass were lower for males raised on cactus pads with unripe fruit or without fruit compared with those raised on cactus pads with ripe fruit (Table 2). The low heritability and evolvability for males raised on cactus pads with unripe fruit and without fruit seem to be driven by low *V*_A_. These findings may indicate that in this system, additive genetic variation for these traits is only expressed when juveniles develop with sufficient nutrition. Furthermore, the low phenotypic variation for males raised without fruit may suggest that in these environments, males may develop into small males independent of their genetic background. The low heritability, CV_A_, and *I*_A_ in cactus with unripe fruit and without fruit developmental environments may indicate a reduction in the ability of these traits to respond to selection in poor nutritional environments. Ripe fruit is rare (Fig. 2), and so, despite their likely importance in mating success (Procter et al. 2012), femur and testis size may be slow to evolve.

Trait heritability has generally been shown to be lower when measured in the field than in the laboratory (e.g., Aspi and Hoikkala 1993; Simons and Roff 1994; Hoffmann 2000; but see Weigensberg and Roff 1996) likely due to the increased environmental variance in the field (Blanckenhorn 2002). Previous studies have shown male reproductive traits to be heritable (e.g., Simons and Roff 1994; Pitnick and Miller 2000; Ward 2000; Simmons and Kotiaho 2002; Simmons 2003), but many of these studies were conducted in laboratories with artificial diets where total phenotypic variation may be reduced. Using the realistic nutritional environments, this experiment may be more applicable to conditions *N. femorata* experiences in the field. Interestingly, phenotypic variation was highest for males raised with ripe fruit. While some of this high phenotypic variation is due to the increased additive genetic variation in the ripe fruit environment, it may also indicate that not all ripe fruits are the same. The quality of ripe cactus fruit may be highly variable compared to unripe fruit and cactus pads.

Cactus fruit availability and quality change seasonally during the time juvenile *N. femorata* develop (Fig. 2, Gillespie et al. 2014). Ripe fruit is rare, and so most juveniles likely develop on unripe fruit or without fruit. Thus, the majority of *N. femorata* males likely do not have the opportunity to reach the larger sizes of males raised on ripe fruit. We found low levels of the additive genetic variation for males that developed on cactus with unripe fruit and without fruit. This result suggests that even if these traits are important for male reproductive success, they may not respond strongly to selection. Interestingly, femur area had low heritability in every developmental environments. Male weapons have been shown to be strongly condition‐dependent in many systems and thus may be particularly sensitive to the environmental conditions (e.g., Moczek and Emlen 1999; Gotoh et al. 2012). Our result of low heritability in femurs is consistent with these previous studies.

Intriguingly, males raised on cactus pads with unripe fruit had the largest femurs but the smallest testes for their body size (Fig. 6). Thus, changes in resource quality did not result in simple differences in size, but instead appears to change the relative trait investment. This finding may be indicative of a priority rule shift in the allocation of resources for these males; that is, males raised on unripe fruit may increase the size of their femurs at the expense of their testes. Such resource allocation shifts have been shown to be induced through dietary changes in a number of other systems (e.g., Basolo 1998). Femur size is an important component of male–male competition, and behavioral assays indicate that males raised on unripe fruit are just as likely to be dominant as males raised on ripe fruit when competing for territories (C. W. Miller, unpubl. data). Because females are attracted to high‐quality territories, these dominant interactions may lead to an increase in mate acquisition for these males. However, the smaller testes of males that developed on the unripe fruit may hinder the lifetime reproductive output if males are sperm limited. Unpublished data suggest that females mated to young adult males that developed on cactus with unripe fruit produce fewer eggs over a 2‐week period than when mated to young adult males that developed on cactus with ripe fruit or without fruit (C. W. Miller, unpubl. data). Low egg production could be a product of sperm limitation, or females could just be less willing to mate with males raised with unripe fruit. An experiment that compares the lifetime reproductive success of males from different developmental environments would be needed to sort this out. It would be particularly interesting to follow offspring production over time to determine whether males can compensate for a bad developmental environment by feeding later in life on high‐quality fruit and boosting sperm production. All males in this study had access to ripe fruit after becoming adults, yet differences in testes mass persisted the 4–5 weeks after eclosion, suggesting these developmental effects may persist throughout adulthood.

In this study, we have found that the nutritional environment during development plays a large role in the expression of sexually selected traits in the leaf‐footed cactus bug, *Narnia femorata*. While we found some evidence that these traits may be heritable in an optimal environment, we found that the nutritional environment plays an overwhelming role in weapon and testis development in this system. As the majority of juveniles in our population develop without ripe fruit (L. Cirino, unpubl. data), there may not be sufficient additive genetic variation expressed in the most common environments for these traits to respond quickly to selection. We found evidence that males raised in the unripe fruit environment may be allocating resources to their femurs at the expense of their testes (Fig. 6). Preliminary data suggest that this allocation strategy may affect their precopulatory and postcopulatory reproductive success, but more specific experiments are needed to examine the role of resource allocation on reproductive success in this system. Finally, the use of nutritionally relevant environments has provided insights into what these animals experience in the field. As sexually selected traits may be particularly sensitive to an animal's condition (Rowe and Houle 1996), we suggest that future laboratory studies on trait heritability and expression would be well served to use, whenever possible, similarly realistic dietary nutrition.

## Conflict of Interest

None declared.
